# Exercise FITT‐V in pregnancy with obesity: Preliminary findings for infant adiposity and intergenerational obesity risk

**DOI:** 10.14814/phy2.70765

**Published:** 2026-02-09

**Authors:** Alex Claiborne, Filip Jevtovic, Ericka M. Biagioni, Lindsey Rossa, Caitlyn Ollmann, Donghai Zheng, Cody Strom, Breanna Wisseman, Samantha McDonald, Edward Newton, Steven Mouro, James DeVente, George A. Kelley, Joseph A. Houmard, Nicholas T. Broskey, Linda E. May

**Affiliations:** ^1^ Department of Kinesiology, Nutrition, and Health Miami University Oxford Ohio USA; ^2^ Department of Translational Neuroscience, Division of Molecular Medicine Wake Forest University School of Medicine Winston Salem North Carolina USA; ^3^ Department of Kinesiology East Carolina University (ECU) Greenville North Carolina USA; ^4^ Human Performance Laboratory ECU Greenville North Carolina USA; ^5^ East Carolina Diabetes & Obesity Institute ECU Greenville North Carolina USA; ^6^ Department of Biomedical Sciences Quinnipiac University Hamden Connecticut USA; ^7^ Department of Human Performance Minnesota State University Mankato Minnesota USA; ^8^ Department of Kinesiology University of Rhode Island Kingston Rhode Island USA; ^9^ School of Kinesiology and Recreation Illinois State University Normal Illinois USA; ^10^ Department of Obstetrics & Gynecology ECU Greenville North Carolina USA; ^11^ School of Public and Population Health Boise State University Boise Idaho USA; ^12^ School of Kinesiology Boise State University Boise Idaho USA

**Keywords:** adiposity, birthweight, dose response, obesity, prenatal exercise

## Abstract

Prenatal exercise decreases offspring adiposity, but it is uncertain whether this relationship is present in offspring exposed to obesity in utero. We aimed to determine whether exercise during pregnancy reduces infant cellular and whole‐body adiposity in offspring born to women with obesity. This is a sub‐analysis of a randomized controlled trial, where women were randomized to supervised exercise or control for ~24 weeks during pregnancy. Exercise FITT‐V metrics (frequency, intensity, time, type, and volume) were collected. Infant mesenchymal stem cells (MSCs) (healthy weight [*n* = 16], obesity [*n* = 21]) were adipogenically differentiated and stained for lipid content. Infant body composition was measured at 1 month of age via skinfold. Among women randomized to control, maternal BMI influenced infant adiposity; infants exposed to obesity had higher body fat percentage (*p* = 0.02). Birthweight was negatively correlated with infant body fat; offspring with lower birthweight had higher body fat (*R* ^2^ = 0.38, *p* = 0.03). Maternal weekly exercise volume trended toward negative association with infant body fat (*R* ^2^ = 0.33, *p* = 0.06) and lipid content (*R* ^2^ = 0.21, *p* = 0.06). For infants born to women with obesity, exercise during pregnancy helps reduce adiposity.

## 1 INTRODUCTION

Obesity (OB) elevates cardiometabolic disease risk and mortality; the early onset of OB in childhood can be a major contributor to risk later in life (Dietz, 1994). Alarmingly, 35 million children under the age of five are currently classified as overweight or OB, which will potentiate the continuation of the global obesity epidemic (GBD 2021 Risk Factors Collaborators, 2021; WHO, 2025). Emerging work has demonstrated the inheritability of OB in offspring with models using infant mesenchymal stem cells (MSCs) isolated from the umbilical cord at birth (Boyle et al., 2016; Erickson et al., 2021; Gyllenhammer et al., 2023). For instance, MSCs harvested from infants born to mothers with OB exhibit increased cellular lipid deposition (Gyllenhammer et al., 2023), alongside higher whole‐body fat percentage (BF%), supporting that OB‐related molecular programming begins in utero (Keleher et al., 2023). Therefore, the MSC model has implications as a tool to study the influence of modifiable lifestyle behaviors in pregnancy, as a way to mitigate infant OB risk (Ana et al., 2025).

Prenatal exercise is a modifiable lifestyle behavior that has been shown to positively influence infant body composition (Clapp, 1996; Clapp et al., 1998; Clapp & Capeless, 1990; McDonald et al., 2020, 2021) and therefore, could be utilized to reduce offspring OB risk, especially in at‐risk pregnancies. For example, exercise during gestation could impart a phenotypic shift in offspring, which later translates to reduced childhood and, therefore, adulthood, OB risk and associated comorbidities (Barker, 2004; Krassovskaia et al., 2022). Given prior findings of higher lipid accumulation in offspring born to mothers with OB (Ana et al., 2025; Boyle et al., 2016), yet lower infant BF% in those exposed to exercise in utero (Clapp & Capeless, 1990; McDonald et al., 2020; McDonald et al., 2021), it could be that maternal exercise mitigates the potential for intergenerational transmission of OB to offspring (Claiborne et al., 2023; Jevtovic et al., 2024; Jevtovic & May, 2023; Krassovskaia et al., 2022). Aerobic exercise increases energy demand, thereby theoretically reducing the storage of excess adipose tissue in adults (Chiu et al., 2017). However, large studies corroborating this notion in offspring exposed to prenatal exercise are lacking (Jevtovic & May, 2023). Furthermore, an assessment of different types and doses of exercise, that is, exercise FITT‐V: frequency, intensity, time, type, and volume, is lacking, despite promising insights from past reviews (Claiborne et al., 2023). Along those lines, previous data has shown that exercise helps regulate maternal weight gain during pregnancy (McDonald et al., 2016), as well as improved birth outcomes and decreased offspring BF% (Claiborne, Wisseman, et al., 2024a; Claiborne, Wisseman, et al., 2024b; Clapp & Capeless, 1990; McDonald et al., 2020); however, to date, only one preliminary study from our group has tested this hypothesis from a dose‐dependent, cellular to whole‐body perspective in offspring (Claiborne, Jevtovic, et al., 2024), and a BMI‐stratified analysis is yet to be performed, despite the intergenerational risk in high‐BMI pregnancies (O'Reilly & Reynolds, 2013).

Based on the preliminary findings from this study (Claiborne, Jevtovic, et al., 2024), the current study aimed to investigate the strength of the relationship between prenatal exercise and infant adiposity, with a dose–response component, in the context of maternal OB. Specifically, this investigation also aimed to highlight the extent of this exercise effect in healthy‐weight and individuals with OB. We hypothesized a significant influence of exercise dose on cellular and whole‐body adiposity of exercise dose in offspring born to women with OB. Testing of this hypothesis is critical for women with OB, whose offspring may require exposure to higher exercise dose to normalize infant adiposity.

## 2 METHODS

### 2.1 Study participants

The current study is a secondary analysis of infant cellular outcomes and infant body composition at 1 month of age, derived from a larger project focused on the influence of maternal exercise types on maternal and infant health outcomes; additionally, this study is a follow‐up to a previously published preliminary report (Claiborne, Jevtovic, et al., 2024). This investigation focused on the comparison of data from offspring born to women with a healthy BMI (<25 kg/m^2^) and women with OB (BMI >30 kg/m^2^). Data for the current study were combined from two prospective, randomized control trials (RCTs) examining the influence of prenatal exercise type on maternal and infant outcomes. The primary focus of this sub‐analysis was to examine measures of body composition and adipogenic MSC lipid accumulation in response to prenatal exercise in offspring born to women with OB. We also investigated the influence of prenatal exercise FITT‐V metrics: frequency, intensity, time, type, and volume. Women enrolled in these studies met the following criteria: clearance from a health care provider to participate in physical activity; between 18 and 40 years of age; pre‐pregnancy body mass index (BMI) >18.5 kg/m^2^; singleton pregnancy; ≤16 weeks gestation; no current alcohol or tobacco use. For this secondary analysis and comparison purposes, we selected data from participants with either a healthy BMI or meeting criteria for OB. Criteria for exclusion included smoking, known pre‐existing conditions (i.e., diabetes mellitus, hypertension, cardiovascular disease, and comorbidities, systemic lupus erythematosus), and/or medications known to affect fetal growth and development.

### 2.2 Ethics statement

This study used study records and umbilical cord mesenchymal stem cells (MSC) collected from participants enrolled in two RCTs investigating the influence of different maternal exercise types on infant outcomes (ClinicalTrials.gov Identifier: NCT03517293 and NCT03838146). Approval for these studies was obtained from the East Carolina University (ECU) Institutional Review Board. Written informed consent was obtained from each participant upon enrollment. All experimental procedures were conducted at ECU.

### 2.3 Pre‐intervention exercise testing & randomization

After study enrollment, participants completed a submaximal treadmill test to determine cardiorespiratory fitness and calculate target heart rate (THR) range for moderate‐intensity training. Peak oxygen consumption (VO_2_ peak) was estimated via the modified Balke protocol previously validated for pregnant women (Mottola, 2008). Participants exercised until they reached 85%–90% of their age‐predicted maximum HR, then VO^2^ was extrapolated to 100% maximum HR. To minimize exposure risk after the start of the COVID‐19 pandemic, women recruited between March 2020 and October 2021 had THR zones for exercise based on their pre‐pregnancy physical activity level and age (Mottola, 2008). Target heart rate zones for exercise components corresponded to maternal HR at 60%–80% of peak oxygen consumption, that is, moderate intensity. Exercise intensity was based off metabolic equivalents (METs), which are calculated from VO2 during steady state exercise. As METs were the chosen metric of intensity, we therefore based our THR zones also off VO_2_, to ensure agreement between the HR measured during each training session, and the calculated MET value for training workload. After receiving clearance from their obstetric provider, participants were randomly assigned to either aerobic exercise (EX), resistance exercise (RE), a combination of both AE and RE (AERE), or an attention‐control (CTRL) group.

### 2.4 Exercise intervention

All participants were supervised by trained exercise instructors in ECU facilities following a standard protocol. All sessions started in early gestation, 12–16 weeks gestation, and women were scheduled three times weekly until delivery (ACOG, 2020). All participant sessions included a 5‐min warm‐up, 50 min of their randomized group activity, and a 5‐min cool‐down.

The AE group completed moderate intensity training on treadmills, ellipticals, recumbent bicycles, rowing, and/or stair‐stepping equipment. Participants were free to switch between equipment, so long as they remained on the chosen piece for at least 10 min. There was no progression in duration of exercise, though exercise intensity progressed dependent on workload at THR. The choice of different types of AE were offered in order to increase compliance. To maintain the appropriate HR zone, speed and grade were adjusted on the treadmill, and resistance and speed levels were adjusted on the other equipment. The RE group completed sessions of three to four sets, aiming for 12 repetitions of each exercise at ~60% of 1 repetition maximum (1‐RM) (ACOG, 2020). Exercises for RE were performed in a circuit with minimal rest (5–10 s) using seated machines (i.e., leg extension, leg curl, shoulder press, chest press, triceps extension, latissimus dorsi pull‐down), dumbbells (i.e., biceps curls, lateral shoulder raises, front shoulder raises), resistance bands, exercise balls, benches, and/or mats. The AERE group performed half of the aerobic protocol and half of the resistance protocol exercises in five circuits, lasting 4.5–5 min each. Resistance exercises were performed, aiming for 12 repetitions (same exercises and equipment as the RE group), while aerobic exercises were performed on the same equipment as the AE group.

To ensure proper intensity was achieved during exercise sessions, the Borg rating scale for perceived exertion (RPE 6–20) and “talk test” were used (Webster & Aznar‐Laín, 2008). Heart rate monitoring (Polar FS2C, Kempele, Finland) ensured appropriate target HR ranges were maintained; target HR zones validated for pregnant women were utilized (Mottola, 2008).

### 2.5 Prenatal exercise dose

Exercising women were asked to complete 3 sessions per week, for 50 min per session, at moderate intensity. Participants were asked to refrain from external exercise throughout the course of the study. To control for differences in duration of pregnancy and start of intervention, exercise dose was analyzed from 16 to 36 weeks gestation for all participants. Frequency was calculated as the number of times the participant attended supervised exercise each week and is expressed as sessions per week. Intensity was calculated based on the published compendium of physical activity (Ainsworth et al., 2011) for each specific exercise performed in supervised sessions. The average weekly intensity (METs) was calculated via the average of these METs through all supervised exercise sessions. Average weekly time in supervised exercise sessions was determined by exercise duration, in minutes for each week, with average duration reported as min/week. Lastly, prenatal exercise volume for all supervised sessions was calculated by (1) multiplying average intensity (METs) by the average weekly duration for volume in MET▪min/week (weekly exercise volume), and (2) multiplying weekly MET▪min by the total number of weeks prenatal exercise was performed through pregnancy for total MET▪min (total pregnancy volume).

### 2.6 Maternal measurements

Maternal pre‐pregnancy height and weight, age, race and ethnicity, gravida, and parity were abstracted from various sources including pre‐screening eligibility and postpartum questionnaires as well as maternal and neonatal electronic health records. Maternal pre‐pregnancy BMI was calculated as [weight (kg)]/[height (m)^2^].

### 2.7 MSC isolation

Isolation of human infant MSCs from umbilical cord explants was completed as previously described (Jevtovic, Lopez, Zheng, et al., 2023; Jevtovic, Zheng, Houmard, et al., 2023; Jevtovic, Zheng, Lopez, et al., 2023). The umbilical cord explants were incubated in mesenchymal growth media, which was made from low glucose DMEM (#11885084, Gibco laboratories, Grand Island, NY) supplemented with 10% MSC‐qualified fetal bovine serum (FBS, #12‐662‐029, Gibco, Thermo‐Fisher Scientific, Waltham, MA, USA), and 1× Gentamicin Amphotericin (Life Technologies, Gaithersburg, MD), until cells reached 80%–100% confluency, then cryopreserved in liquid nitrogen for later experimentation, in which they were thawed and re‐seeded for adipogenic induction.

### 2.8 Adipogenic induction

MSCs were cultured in mesenchymal growth media in 6‐well plates (#10062‐892) or T‐75 flasks (#10062‐860, VWR International, Radnor, PA, USA) until 80%–90% confluency. To reduce the potential loss of the infant phenotype, all experiments were performed in similar MSC passages (Boyle et al., 2016; Keleher et al., 2023). Adipogenic induction was initialized 2–5 days post‐confluence as previously done (Boyle et al., 2016). Adipogenic induction media (AIM) containing low glucose DMEM (Gibco laboratories, Grand Island, NY) supplemented with 5% heat‐inactivated fetal bovine serum (FBS), 0.1X Penicillin Streptomycin (Life Technologies, Gaithersburg, MD), 1 μM dexamethasone (#D4902, Sigma Aldrich, St. Louis, MO, USA), 0.2 mM indomethacin (#I7378, Sigma Aldrich, St. Louis, MO, USA), 0.5 mM 3‐isobutyl‐1‐methylxanthine (#I5879‐1G, Sigma Aldrich, St. Louis, MO, USA), and 170 nM insulin (Humulin®, Eli Lilly and Company, Indianapolis, IN, USA) was used. Adipogenesis was maintained with adipogenic maintenance media (AMM) containing low‐glucose DMEM (Gibco Laboratories, Grand Island, NY) supplemented with 5% heat‐inactivated fetal bovine serum (FBS), 0.1X Penicillin Streptomycin (Life Technologies, Gaithersburg, MD), and 170 nM insulin. Adipogenesis was performed for 21 days with 3‐day cycles of AIM (3d) − AMM (3d) − AIM (3d) − AMM (3d) − AIM (3d) − AMM (3d) − AMM (3d). All experiments were performed on the 21st day of adipogenic induction.

### 2.9 Lipid storage

For the assessment of neutral lipid content, cells were rinsed with Dulbecco's Phosphate Buffered Saline (DPBS), fixed with 4% paraformaldehyde, and stained using 0.2% Oil Red O stain (ORO; #A12989.22, Thermo‐Fisher Scientific, Waltham, MA, USA) in propylene glycol (Boyle et al., 2016). Thereafter, cells were rinsed with 85% propylene glycol and deionized water. ORO stain was solubilized with isopropanol and transferred to a clean 96‐well plate (#10861‐666, VWR International, Radnor, PA, USA) and analyzed via spectrophotometry at 520 nm for the degree of ORO staining. To account for cell density, each well was then stained with 0.3% Janus Green B (#A17391.14, Thermo‐Fisher Scientific, Waltham, MA, USA), which provided a general cell count on each well. After rinsing with deionized water, cellular Janus Green was solubilized with 0.5 M HCl; samples were analyzed with spectrophotometry at 595 nm for the degree of Janus Green staining, that is, cell count. Cellular ORO stain was then normalized to cellular Janus Green stain to estimate the lipid content per cell. All staining was done with five replicates and included one no‐cell control well.

### 2.10 Infant measurements

Birth measurements (weight, length, ponderal index, BMI, 1 and 5‐min Apgar scores) and infant sex were extracted from neonatal electronic health records. At 1 month of age, infant weight (kg), length (m), BMI (kg/m^2^), and BF% were measured by trained staff in a designated pediatric clinic as previously published (Strom et al., 2022). Weight and length were measured using a standard, calibrated infant scale and horizontal stadiometer, respectively. Skinfold thickness was measured via standard Lange calipers (BETA Technologies, Burlington, VT, USA) at three designated anatomical sites on the right side of the infant's body: biceps, triceps, and subscapular. Values for these sites were summed to determine total skinfold thickness. The skinfold thickness data were then used to calculate BF% using the following equation by Slaughter et al. 38: Body Fat % = (1.21 × ([triceps] + [subscapular])) − ((0.008) × ([triceps] + [subscapular]) × ([triceps] + [subscapular])) − 1.7.

### 2.11 Statistical analysis

To assess the influence of maternal BMI status, that is, HW and OB, independent samples *t*‐tests were run for infant BF% between maternal BMI status. To investigate possible reasons for these relationships, we examined birth outcomes in these women. Specifically, independent samples *t*‐tests were run to test for differences in birthweight between groups, and then Pearson product–moment correlations were run between birthweight and 1‐month BF%. Analysis of covariance (ANCOVA) was performed to compare the independent effects of obesity and exercise dose on infant body fat %. For non‐parametric data, for example, gravida and parity, group comparisons were made with Kruskal–Wallis. Finally, Pearson product–moment correlations were run between maternal exercise FITT‐V throughout pregnancy and adiposity outcomes, including MSC lipid content and 1‐month BF%, independently for HW and OB offspring. A two‐tailed alpha level ≤0.05 was considered statistically significant. All statistical analyses were completed using SPSS software (version 28.0.1.1, SPSS Inc., IBM Corp., Chicago, IL).

## 3 RESULTS

Comparisons were made based on a total sample size of 37 (*n* = 16 HW, *n* = 21 OB) adipogenically differentiated umbilical cord MSCs. Infant body composition, skinfolds, and BF% data were available at 1 month of age for *n* = 31 infants (*n* = 16 HW, *n* = 15 OB). Maternal characteristics were similar between exercise and control groups (Table 1). Women averaged a total of 22 weeks of the intervention. There was no difference in attendance between HW and OB (*p* = 0.45). From a standardized period of 16–36 weeks gestation, exercise volume ranged between 180 and 750 MET▪min/week, spread over 14–74 total sessions, and resulting in 3000–17,500 total MET▪minutes through pregnancy.

**TABLE 1:** Maternal and infant characteristics.

Maternal	HW (*n* = 16)	OB (*n* = 21)	*p*
% in EX group	88	76	0.40
pre‐Pregnancy BMI, kg▪m^−2^	22.1 ± 1.7	35.5 ± 4.3	**<0.01**
Age years	30.6 ± 4.4	29.8 ± 5.4	0.64
% BIPOC	13	38	0.09
Gravida^@^	1 (1,3)	1 (1,4)	0.38
Parity^@^	0 (0,2)	0 (0,2)	0.57
VO_2_peak, mL▪kg▪min^−1^	22.5 ± 4.6	18.8 ± 4.0	0.04

Infant	HW (*n* = 16)	OB (*n* = 21)	*p*
% Female	50	40	0.58
GA at Birth, weeks	39.8 ± 0.7	39.1 ± 1.1	0.05
% C‐Section	7	45	**0.01**
Birth length, m	0.5 ± 0.0	0.5 ± 0.0	0.93
Birthweight, kg	3.5 ± 0.3	3.6 ± 0.5	0.63
Birth BMI, kg▪min^−1^	13.9 ± 1.3	14.2 ± 1.8	0.62

*Note*: Data reported as mean ± SD. Non‐parametric data (gravida, parity) reported as median (min, max). Maternal characteristics measured before commencement of exercise (12–16 weeks of gestation) or documented at birth (pregnancy complications). Infant characteristics measured at birth. GA gestational age, C‐Section Caesarean Section. ^@^Non‐parametric data, groups compared with Kruskal–Wallis. Values in bold indicate significance at *p* < 0.05.

Abbreviations: BIPOC, Black or Indigenous People of Color; BMI, body mass index; CON, control; EX, exercise; kg, kilograms bodyweight; m, meters height.

**FIGURE 1:**
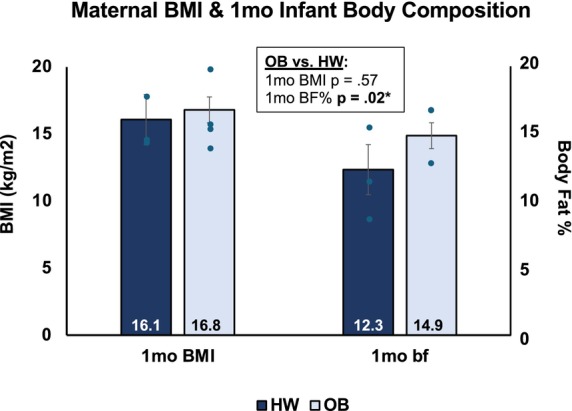
Maternal BMI and 1‐month infant body composition. In control participants only, infant body fat percentage (BF%) at 1 month of age varied based on maternal BMI status (healthy weight HW or obese only OB), while this sample showed no difference in BMI. This shows an influence of maternal BMI independent of exercise.

**FIGURE 2:**
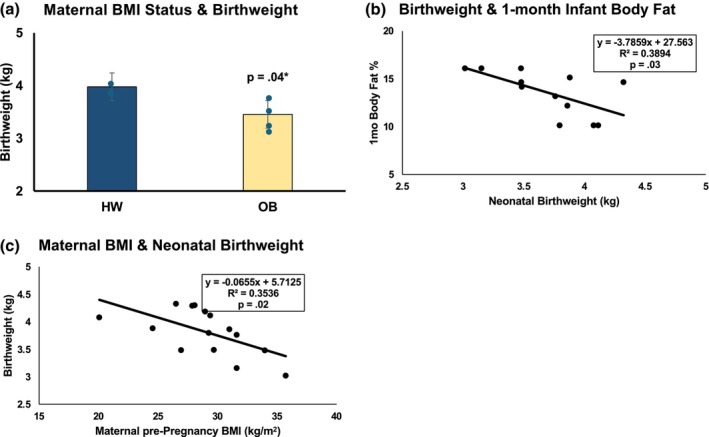
Offspring birthweight and whole‐body adiposity: CON Only. (a) In control participants only, offspring birthweight was significantly lower in those born to women with OB compared to HW. (b) Maternal BMI as a continuous variable was negatively correlated with birthweight, such that women with higher BMI pre‐pregnancy gave birth to smaller neonates. (c) Birthweight showed a strong, negative association with infant 1‐month BF%, such that neonates born with lower birthweight also showed a higher relative contribution of adipose tissue.

Exposure to OB in utero revealed a significant increase in incidence of Caesarean section (Table 1), but no other statistically significant effects when exercise and control subjects were pooled together and grouped based on BMI (*n* = 16 HW, *n* = 21 OB). However, among control participants, morphometric differences were found at birth and 1 month of age between offspring born to women with HW or OB (Figures 1 and 2). Maternal BMI influenced 1‐month BF%, such that offspring exposed to OB had significantly higher BF% at 1 month of age (*p* = 0.02, Figure 1), despite no significant difference in BMI at this age (*p* = 0.57, Figure 1). Furthermore, women with OB gave birth to offspring with 500 g (13%) reduced birthweight compared to HW (*p* = 0.04, Figure 2a). As a reflection of this observation, maternal BMI was negatively associated with neonatal birthweight (*R* ^2^ = 0.35, *p* = 0.02, Figure 2b), where higher maternal BMI was associated with lower birthweight. This relationship appeared to have implications for offspring through 1 month of age, as birthweight was negatively correlated with infant BF% at 1 month, such that offspring with lower birthweight had higher BF% at 1 month (*R* ^2^ = 0.38, *p* = 0.03, Figure 2c).

**FIGURE 3:**
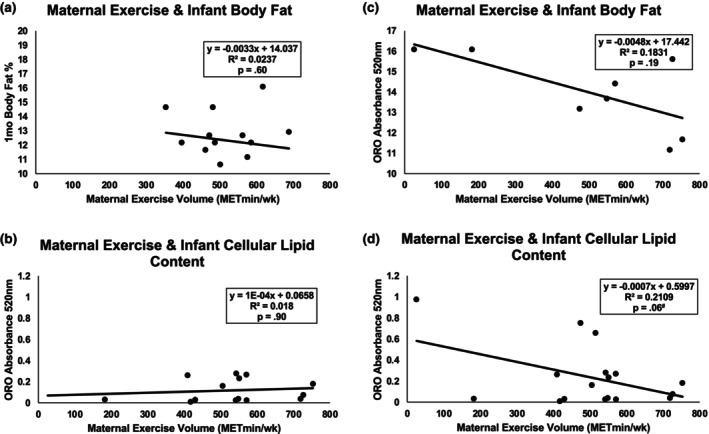
Effect of prenatal exercise on offspring cellular & whole‐body adiposity in OB versus HW pregnancies. (a) For HW only, no significant relationship was found between maternal exercise volume per week and infant BF%, while (b): Exercise also did not influence cellular lipid content. (c) For OB only, weekly MET × minutes of exercise during pregnancy were strongly, yet non‐significantly associated with infant BF%. (d) For OB only, there was a strong association between maternal exercise volume and infant cellular lipid content, which trended close to statistical significance.

To compare the potential for exercise to modify the relationship between high maternal BMI and high infant BF%, we tested for relationships between maternal exercise dose and infant BF% independently in HW and OB groups (Figure 3). In HW, no significant associations were found between maternal weekly exercise volume (MET × min/week) and infant BF% (*R* ^2^ = 0.02, *p* = 0.60, Figure 3a) or infant cellular lipid content (*R* ^2^ = 0.01, *p* = 0.90, Figure 3b). However, in OB, maternal weekly exercise volume tended to be negatively associated with infant BF% (*R* ^2^ = 0.33, *p* = 0.06, Figure 3c) and infant cellular lipid content (*R* ^2^ = 0.21, *p* = 0.06, Figure 3d). To compare the independent effects of obesity and exercise dose on infant body fat %, ANCOVA revealed a significantly higher body fat % in OB versus HW (*p* = 0.004), as well as a trend for lower body fat % in offspring exposed to longer duration prenatal exercise (Table 2; *p* = 0.07).

**TABLE 2:** ANCOVA: effect of obesity and exercise dose on 1‐month infant body fat %.

Model	*B*	Std. error	Lower 95%	Upper 95%	*p* value
1‐month infant body fat %		*p* value = 0.011, mean square = 111.8, *F* = 4.736			
Obese or HW	−2.772	0.864	−4.568	−0.975	**0.004**
Average exercise intensity (METs)	−0.906	0.744	−2.452	0.641	0.237
Average weekly exercise duration	−0.029	0.015	−0.061	0.003	0.073

*Note*: Value in bold indicates significance at *p* < 0.05.

## 4 DISCUSSION

The current study aimed to investigate the strength of the dose‐dependent relationship between prenatal exercise and infant adiposity in offspring born to OB versus HW pregnant individuals. We hypothesized that offspring born to women with OB would demonstrate higher BF%, when compared to those born to HW mothers, and for this to be reflected by increased lipid storage in infant MSCs (Claiborne, Jevtovic, et al., 2024) In contrast, we also hypothesized that the effect of exercise would be stronger, that is, a stronger negative association, between exercise dose and lower BF% and lipid storage in OB offspring. Based on our preliminary findings (Claiborne, Jevtovic, et al., 2024), it was expected that this reduction in adiposity would be due to higher time‐component metrics of exercise dose, as opposed to the intensity of exercise. Our findings support these hypotheses, with OB offspring showing significantly higher BF% at 1 month of age. Maternal exercise weekly volume (MET × min/week) showed a stronger relationship with whole‐body and cellular adipose measures in OB offspring, compared to HW. Of clinical importance, infant BF% was up to 4 points lower in high‐volume exercisers, with all infant BF% within normal range for previously reported data from birth to 6 weeks of age, that is, ~10%–20% (Carberry et al., 2010).

Our findings show a 2–3‐point higher BF% at 1 month of age in infants born to women with OB, when compared with HW women. This is despite a non‐significant difference in infant BMI at this time point, suggesting that adipose tissue accumulation occurs in the absence of excessive weight gain, thereby lowering lean mass. The relationship between maternal and offspring adiposity has previously been suggested (Castillo‐Laura et al., 2015; O'Reilly & Reynolds, 2013), and our assessment at 1 month of age suggests this relationship existed before the confounding influence of external environment exposures after delivery. As we utilized a three‐site skinfold model to estimate infant BF% (Freedman et al., 2013; Slaughter et al., 1988) we were able to identify those regions that showed relatively higher adiposity. We found BF% to be elevated in OB due to slightly larger skinfold measurements at the triceps site. Interestingly, however, our preliminary data from this project show that subscapular skinfold measurement is reduced as a result of prenatal exercise exposure (Claiborne, Jevtovic, et al., 2024). Future work should corroborate changes in skinfold data against BF% measurements taken from more up‐to‐date technology such as dual‐energy x‐ray absorptiometry (DXA).

In addition to the above, we demonstrated that in cases where the mother meets the minimum American College of Obstetricians and Gynecologists (ACOG) recommendations for exercise volume, that is, MET × minutes, per week, infants showed lower BF% at 1‐month. This relationship was found for infants born to OB women but not women with HW, despite our previous finding of a significant relationship in a pooled preliminary data set (Claiborne, Jevtovic, et al., 2024). The current study reinforced that this dose‐dependent change in adiposity occurs at the cellular level, as we found maternal weekly exercise volume to correlate with triglyceride content in MSCs. This supports our preliminary work, but the current study added a BMI‐stratified perspective in a larger data set. In essence, we found the absence of an effect on cellular lipid content in HW participants. We also note that the presence of an effect in offspring exposed to OB in utero suggests the following: (1) that the adipogenic MSC model reflects individual trends in infant BF% at 1‐month, and (2) that higher weekly exercise volume is especially important in this condition.

This study found maternal OB to also influence birthweight as a possible explanation for the higher BF% measurement at 1 month of age. Earlier studies have examined this relationship (Gaillard et al., 2014; Gul et al., 2020), and despite suggestions that higher birthweight is to be expected in OB pregnancies (ACOG, 2013), birthweight was actually lower in this cohort of women with OB. Additionally, reduced birthweight was negatively correlated with 1‐month infant BF%, suggesting that low birthweight offspring could accumulate more adipose tissue during development. This suggests that women with OB prior to and during pregnancy should consider modifiable health behaviors shown to reduce infant adipose tissue accumulation. We have previously shown that exercise during pregnancy, especially at higher doses, is beneficial to cellular lipid accumulation, and this translates to lower BF% (O'Reilly & Reynolds, 2013). Other work from our laboratory suggests that higher energy expenditure in offspring at rest, as well as more lipid oxidation, as possible mechanisms (Jevtovic et al., 2024; Jevtovic, Zheng, Lopez, et al., 2023). The current study adds to a growing body of work demonstrating that higher exercise doses are especially important in women with OB in order to reduce OB risk in offspring.

The major strengths of this study include the supervised exercise intervention with pregnant women exercising for >20 weeks throughout pregnancy. Additionally, we were able to calculate weekly and total pregnancy exercise dose from sessions. To the best of the investigative team's knowledge, this is the first assessment of how supervised exercise interventions influence offspring adiposity in offspring exposed to OB in utero. In addition to strengths, we acknowledge several potential limitations. For example, because this study focused specifically on OB participants, for which we had fully collected and analyzed cellular markers of adiposity, we had a small sample size. The sample size for adipogenic differentiation was limited to the availability of clinical data for comparison (infant adiposity measures, birth outcomes, and complete maternal FITT‐V exercise metrics) in subjects with cryopreserved umbilical MSC samples. The former notwithstanding, we believe this data will serve an important role for future studies. Finally, this sample consisted of otherwise healthy pregnancies, and while a focus was on OB, our investigation does not reflect these relationships in women with other chronic cardiometabolic conditions, such as gestational diabetes.

## 5 CONCLUSION

While previous data from our group has shown that exercise during pregnancy influences offspring adiposity, no investigation to date has targeted offspring exposed to OB in utero. A plethora of data support the developmental origins of health and disease, which posits that offspring exposed to OB during pregnancy are at increased risk of developing OB through development, primarily due to the excess accumulation of adipose tissue. Our data reveal that while prenatal exercise reduces BF% and cellular lipid accumulation in offspring, this exercise dose‐dependent relationship is stronger in offspring born to women with OB relative to HW. This and future investigations therefore have large implications for fighting the intergenerational cycle of OB.

## AUTHOR CONTRIBUTIONS

**Alex Claiborne**: visualization, writing—original draft, writing—review and editing, formal analysis, funding acquisition, investigation. **Filip Jevtovic**: data curation, software, writing—review and editing. **Ericka Biagioni**: writing—review and editing. **Lindsey Rossa**: project administration, writing—review and editing. **Caitlyn Ollmann**: project administration, writing—review and editing. **Donghai Zheng**: project administration, writing—review and editing. **Cody Strom**: data curation, software, writing—review and editing. **Breanna Wisseman**: data curation, software, writing—review and editing. **Samantha McDonald**: data curation, software, writing—review and editing. **Edward Newton**: project administration, writing—review and editing. **Steven Mouro**: project administration, writing—review and editing. **James deVente**: project administration, writing—review and editing. **George A. Kelley**: methodology, writing—review and editing. **Joseph A. Houmard**: conceptualization, methodology, writing—review and editing. **Nicholas T. Broskey**: conceptualization, resources, writing—review and editing. **Linda E. May**: conceptualization, data curation, funding acquisition, investigation, methodology, project administration, resources, software, supervision, validation, writing—review and editing.

## FUNDING INFORMATION

Thrasher Research Foundation grant #02154 (PI: Claiborne), American Heart Association grants #15GRNT24470029 (PI: May), #18IPA34150006 (PI: May) and NIH #5R01DK129480‐01 (PI: May), NIH R01DK137945 (PI: Broskey), as well as internal funds provided by East Carolina University. Curation of funds only. Funding sources were not involved in study design or the collection, analysis, and interpretation of data.

## CONFLICT OF INTEREST STATEMENT

The authors report no conflict of interest.

## ETHICS STATEMENT

This study used study records and umbilical cord mesenchymal stem cells (MSC) collected from participants enrolled in two RCTs investigating the influence of different maternal exercise types on infant outcomes (ClinicalTrials.gov Identifier: NCT03517293 and NCT03838146). Approval for these studies was obtained from the East Carolina University (ECU) Institutional Review Board. Written informed consent was obtained from each participant upon enrollment. All experimental procedures were conducted at ECU.

## CLINICAL TRIAL REGISTRATION

ClinicalTrials.gov Identifier: NCT03517293; NCT04805502.

## Data Availability

Data generated/analyzed during the current study is available upon request from the corresponding/senior author.
